# Preventive Effects of Ginkgo-Extract EGb 761^®^ on Noise Trauma-Induced Cochlear Synaptopathy

**DOI:** 10.3390/nu14153015

**Published:** 2022-07-22

**Authors:** Konstantin Tziridis, Holger Schulze

**Affiliations:** Experimental Otolaryngology, ENT Clinic Head and Neck Surgery, University Hospital Erlangen, 91054 Erlangen, Germany; konstantin.tziridis@uk-erlangen.de

**Keywords:** hearing loss, tinnitus, pharmacological treatment, auditory brainstem response, gap prepulse inhibition of the acoustic startle response, immunohistology, synaptopathy, rodents

## Abstract

Noise trauma-induced loss of ribbon synapses at the inner hair cells (IHC) of the cochlea may lead to hearing loss (HL), resulting in tinnitus. We are convinced that a successful and sustainable therapy of tinnitus has to treat both symptom and cause. One of these causes may be the mentioned loss of ribbon synapses at the IHC of the cochlea. In this study, we investigated the possible preventive and curative effects of the *Ginkgo biloba* extract EGb 761^®^ on noise-induced synaptopathy, HL, and tinnitus development in Mongolian gerbils (*Meriones unguiculatus*). To this end, 37 male animals received EGb 761^®^ or placebo orally 3 weeks before (16 animals) or after (21 animals) a monaural acoustic noise trauma (2 kHz, 115 dB SPL, 75 min). Animals’ hearing thresholds were determined by auditory brainstem response (ABR) audiometry. A possible tinnitus percept was assessed by the gap prepulse inhibition acoustic startle reflex (GPIAS) response paradigm. Synaptopathy was quantified by cochlear immunofluorescence histology, counting the ribbon synapses of 15 IHCs at 11 different cochlear frequency locations per ear. We found a clear preventive effect of EGb 761^®^ on ribbon synapse numbers with the surprising result of a significant increase in synaptic innervation on the trauma side relative to placebo-treated animals. Consequently, animals treated with EGb 761^®^ before noise trauma did not develop a significant HL and were also less affected by tinnitus compared to placebo-treated animals. On the other hand, we did not see a curative effect (EGb 761^®^ treatment after noise trauma) of the extract on ribbon synapse numbers and, consequently, a significant HL and no difference in tinnitus development compared to the placebo-treated animals. Taken together, EGb 761^®^ prevented noise-induced HL and tinnitus by protecting from noise trauma-induced cochlear ribbon synapse loss; however, in our model, it did not restore lost ribbon synapses.

## 1. Introduction

The treatment of tinnitus—the phantom percept of a sound without an external source—is often focused on coping with or suppressing this symptom of hearing impairment rather than the treatment of its causes [1,2,3,4]. This has at least two reasons. First, the neurophysiological mechanisms leading to the percept are still not completely understood and are subject of debate [5,6,7,8,9]. Second, with very few exceptions as in the case of the typewriter tinnitus [10], no effective treatments that can cure tinnitus have been found. Especially in the case of tonal or narrowband tinnitus, accounting for up to 70% of all cases, several approaches have been evaluated, including but not limited to pharmacological therapy [11], notched music or noise therapy [12,13,14], vagus nerve stimulation [15], and hyperbaric oxygen therapy [16]. Most of these approaches did not show strong effects on the tinnitus percept, at least in chronic cases. On the other hand, approaches that focus on the restoration of hearing, e.g., with hearing aids or cochlear implants [17], show good effects at least in some patients. Furthermore, our own approach with an individually tailored near-threshold noise [18,19], which is based on the idea that tinnitus is a byproduct of a physiological mechanism optimizing auditory neuronal information transmission [8,9,20,21,22], showed promising results in tinnitus attenuation. Nevertheless, because of its convenience, pharmacological treatment of both the symptoms and the underlying source would be favorable. In our view, the primary event is damage to neurons of the inner ear [23,24,25], thus affecting the downstream transmission of auditory information from the inner hair cells (IHC) to the central nervous system via their ribbon synapses and the auditory nerve [20].

One candidate for such a pharmaceutical product is the clinically and non-clinically tested *Ginkgo biloba* leaf extract EGb 761^®^ (for a review, see [11]). It has already shown significant effects against the development of hearing loss and tinnitus in either preventive or curative approaches in animal models as assessed with hearing threshold measurements and behavioral tests [26,27]. Until now, it is unknown how exactly the extract prevents noise trauma-induced hearing loss and, subsequently, the development of a tinnitus percept. We here aimed to investigate these questions starting at the level of the cochlear ribbon synapses of Mongolian gerbils. Then, we performed hearing threshold estimations based on auditory brainstem responses (ABRs) and ended with the percept, tested with a reflex-based behavioral paradigm in a preventive and a curative approach. We follow the hypothesis that, if EGb 761^®^ is able to prevent noise trauma-induced synaptopathy of the ribbon synapses of the IHCs, no (hidden) hearing loss should emerge and, therefore, no tinnitus should develop. On the other hand, if synaptopathy cannot be prevented or cured, hearing loss and in some cases tinnitus should emerge.

## 2. Materials and Methods

### 2.1. Ethical Statement

Mongolian gerbils (*Meriones unguiculatus*) were housed in type IV cages in a Uni-Protect air flow cabinet (Zoonlab, Castrop-Rauxel, Germany) in groups of 3–4 animals with free access to water and food at 24 °C room temperature and 50% relative air humidity under a 12/12 h dark/light cycle. The use and care of animals was approved by the state of Bavaria (Regierungspräsidium Mittelfranken, Ansbach, Germany, No. 54.2-2532.1-02/13). A total of 37 10–12 week old male gerbils, purchased from Janvier (Saint Berthevin, France), were used in this study.

### 2.2. Time Regime of Experiments

**Figure 1:**
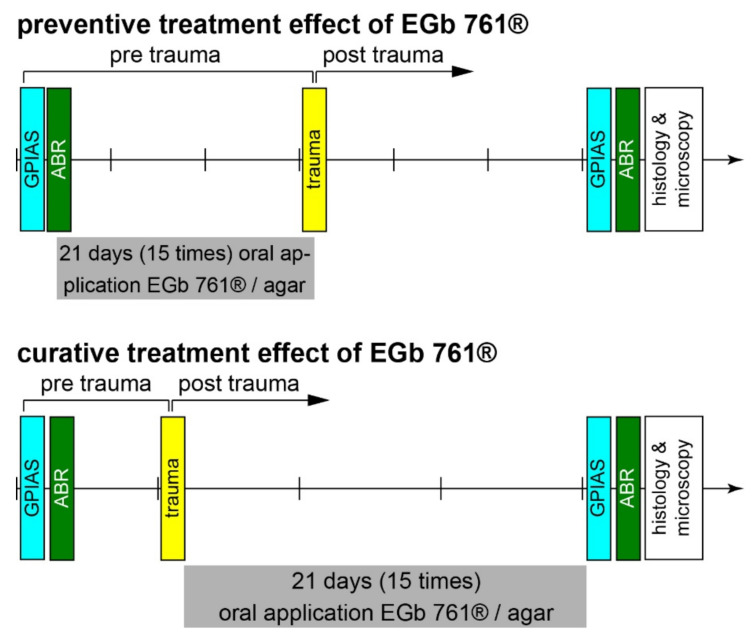
Time regimes of experiments for *preventive treatment effects* (upper panel) and *curative treatment effects* of EGb 761^®^ (lower panel) on hearing thresholds (ABR), behavioral tinnitus assessment (GPIAS), and IHC ribbon synapse counts (immunohistology). The divisions on the time arrow represent 1 week each.

All animals were handled over the time course of 1 week before the beginning of the experiments and accustomed to the setup environments to minimize stress. Animals were separated into four groups with at least eight animals as the minimum number of individuals needed for subsequent statistics. The groups consisted of eight control and eight substance animals in the preventive treatment approach (16 animals in total) and 10 control and 11 substance animals in the curative experiments (21 animals in total). The experimenters were blinded to the content of the medium applied (control or EGb 761^®^). All methods used have been described elsewhere [20,26,27,28,29,30,31,32] and are, therefore, only briefly described below and schematically depicted in Figure 1. Briefly, agar medium was applied orally by 15 daily applications, either 3 weeks before or after a mild monaural acoustic noise trauma. Before any treatment, animals were behaviorally and electrophysiologically evaluated to obtain the baseline conditions for the tinnitus test and the hearing threshold measurements. Three weeks after trauma, the measurements were repeated, the animals were sacrificed, both cochleae were extracted, and ribbon synapse numbers were evaluated immunohistologically.

### 2.3. Agar Medium and Oral Application

The method used here was identical to the method described earlier [26]. EGb 761^®^ is a dry extract from Ginkgo biloba leaves (35 to 67:1), with extraction solvent acetone 60% (*w*/*w*). The extract was adjusted to 22–27% ginkgo flavonoids calculated as ginkgo flavone glycosides, 5.4–6.6% terpene lactones consisting of 2.8–3.4% ginkgolides A, B, and C and 2.6–3.2% bilobalide, 6.5–9.5% proanthocyanidins, and less than 5 ppm ginkgolic acids.

EGb 761^®^ provided by Dr. Willmar Schwabe Pharmaceutics (Karlsruhe, Germany) was diluted in 2% agar in water. The control medium was 2% agar in water. To blind the experimenters to the content of the agar medium, blue food colorant was added. Animals were always treated pairwise, i.e., one received the substance and one the control, without knowledge of the person feeding the animals.

As illustrated in Figure 1 the animals were fed daily with the extract in agar medium (100 mg extract/kg body weight) via a feeding cannula over 3 weeks either before the trauma or over 3 weeks after the trauma (substance groups). Alternatively, they were fed over the same time with the same volume of agar medium only (control groups).

### 2.4. Behavioral Tinnitus Assessment (by GPIAS)

The acoustic startle reflex (ASR) is a widely used behavioral approach that does not require any training prior to testing. It has been modified into gap prepulse inhibition of the ASR (GPAIS) to assess a possible tinnitus percept [30,33,34]. In this study, animals were tested first, with a gap/no-gap paradigm to measure the prepulse inhibition (PPI) of the ASR amplitudes usually 1–2 days before the acoustic trauma. These data were used as the healthy baseline condition for later calculation of the effect size of the change in PPI (see Section 2.8). For ASR measurement, animals were placed in a 15 cm long, 4.2 cm wide acrylic tube on a custom-made 3D acceleration sensor platform in front of two speakers (one for the background stimulus and one for the startle pulse) inside a sound-attenuated, anechoic, dark chamber. For habituation, the animals were allowed 15 min of rest, followed by stimulation with five stimuli that were discarded from further analysis. The proper GPIAS stimuli consisted of bandpass-filtered background noise (10 s; 60 dB SPL) with center frequencies of 1 kHz, 2 kHz, 4 kHz, and 8 kHz and half an octave width each. Each stimulus was presented 60 times, with (30 stimuli) or without (30 stimuli) a 20 ms gap, 100 ms before a startle stimulus (white noise burst, 20 ms duration, 2 ms sin^2^ ramp, 105 dB SPL) in a pseudorandomized manner. The complete behavioral measurement of 240 stimuli had a duration of roughly 45 min. The exact same measurement was repeated 21 days after the monaural acoustic trauma at the same time of the day.

### 2.5. Assessment of Hearing Thresholds (by ABR)

After the GPIAS measurement, the animals were anesthetized with 0.3 to 0.4 mL of a mixture of ketamine and xylazine (ketamine 500 mg/kg, xylazine 25 mg/kg) and placed on a remote-controlled heating pad inside a sound-attenuated, anechoic, dark chamber to obtain individual ABR based audiograms [32]. The stimulation frequencies between 1 and 8 kHz in octave steps for stimulation interval; 500 ms interstimulus interval) were presented. The complete measurement intensities ranging from 0 to 90 dB SPL in 5 dB steps were presented free-field via a loudspeaker placed 3 cm in front of the pinna of the measured ear. For each ear, stimulus, and intensity, 300 repetitions of pairs of 6 ms long (each) phase-inverted double stimuli (100 ms intrastimulus of one ear took less than 30 min.

For ABR measurements, three silver electrodes were placed subcutaneously, one retroaural above the bulla of the tested ear (recording electrode), another one central between both ears (reference electrode), and a last one at the basis of the tail (ground electrode). The signal was recorded and filtered (bandpass filter 400 to 2000 Hz) via a Neuroamp 401 amplifier (JHM, Mainaschaff, Germany). As for the GPIAS, the exact same measurements were repeated 21 days after the monaural acoustic trauma at the same time of the day.

### 2.6. Monaural Acoustic Trauma

In this test, 1–2 days after the baseline measurements of GPIAS and ABR, the animals were again anesthetized with the same ketamine/xylazine mixture and placed on a remote-controlled heating pad, 10 cm in front of a speaker aligned to their left ear inside a sound-attenuated, anechoic, dark chamber. The right ear (intraindividual control) was muted with protective ear foam (Ohropax^®^). A mild monaural acoustic trauma was induced in the left ear using a 2 kHz pure tone at 115 dB SPL lasting for 75 min (e.g., [20]). The animals were usually sleeping for 120 min and were returned to their home cage after fully waking up.

### 2.7. Immunohistology

Whole-mount immunohistology was performed on both cochleae of the animals as described before [20]. Then, 21 days post trauma, after obtaining all behavioral and electrophysiological data, the animals were sacrificed, and the cochleae of the traumatized and non-traumatized (control) ears were extracted and fixed in 4% formaldehyde for 1 h. After decalcification in 0.1 M EDTA for 1–2 days, the cochlear turns were usually cut into three pieces and immunostained for synaptic ribbon protein carboxy-terminal binding protein 2 (CTBP2). Cochlear whole mounts were immunostained overnight at 4 °C with primary antibody against CTBP2 (mouse anti-CTBP2 at 1:200; BD Transduction Labs, Heidelberg, Germany). A secondary antibody (donkey anti mouse conjugated with Cy3, 1:400, Dianova, Hamburg, Germany) was applied for 1 h at room temperature. The whole mounts were then prepared for microscopy, and the ribbon synapses of the IHC were counted at 11 different sites with 15 IHCs each (see below).

### 2.8. Data Evaluation and Statistical Analysis

The obtained data of the GPIAS and ABR measurements were evaluated objectively and automatically using custom-made Python programs [32,34]. Tinnitus development was tested for each animal and frequency individually by *t*-tests of the log-normalized PPI. The log-normalization of the response amplitudes of gap (A_gap_) and no-gap (A_no-gap_) by log(A_gap_/A_no-gap_) is necessary as it has been shown that only after this calculation is parametrical testing (e.g., by *t*-tests) permissible [30]. A significant decrease (*p* < 0.05; Bonferroni-corrected) in the GPIAS-induced change of the ASR amplitude (i.e., PPI) after the trauma (PPI_post_) relative to conditions before the trauma (PPI_pre_) in that test was rated as an indication of a tinnitus percept at that specific frequency. The effect size of the log-normalized PPI change (log(PPI_post_) − log(PPI_pre_)) can be interpreted as a correlate of the subjective tinnitus loudness. With this behavioral approach, we are able to group animals independent of any other measurement into those with behavioral signs of a tinnitus percept and those without (e.g., [20]). The resulting distributions were tested by a chi^2^ test for multiple groups.

The threshold determination was performed by an automated objective approach using the root-mean-square (RMS) values of the ABR amplitudes fitted with a hard sigmoid function using the background activity as the offset [32]. The mean threshold was set at the level of slope change of this hard sigmoid fit independently for each frequency and for each timepoint. Hearing loss (HL) was calculated by subtracting the post-trauma from the pre-trauma thresholds and tested by parametric tests. Positive HL values indicate worse hearing after the trauma, while negative values indicate better hearing. The HL was calculated separately for each ear, i.e., control and trauma ear.

**Figure 2:**
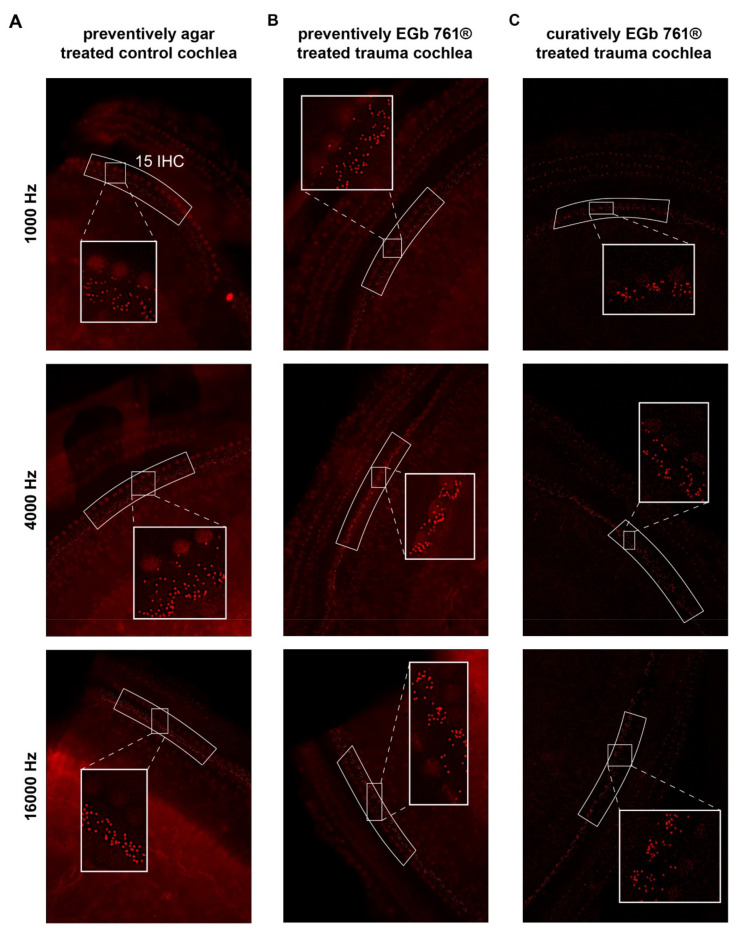
Exemplary immunostained histological slices of three different animals’ cochleae. (**A**) Slices of a preventively agar-treated control cochlea. Given are the tonotopic locations of 1000 Hz (top), 4000 Hz (center) and 16,000 Hz (bottom) with the 15 evaluated IHCs marked in the white arc-box. The insets show a zoom on three of the IHCs with the ribbon synapses in red. (**B**) Slices of a preventively EGb 761^®^-treated trauma cochlea. (**C**) Slices of a curatively EGb 761^®^-treated trauma ear.

The number of synapses per IHC was counted from the microscopic images by eye by one investigator blinded to the status of the cochlea (traumatized/control ear) or any other attribute of the animal. For exemplary histological slices, refer to Figure 2, which shows fluorescence microscope images at 1 kHz, 4 kHz, and 16 kHz locations of a preventively agar-treated control cochlea (Figure 2A), a preventively EGb 761-treated trauma cochlea (Figure 2B), and a curatively EGb 761-treated trauma cochlea (Figure 2C). The frequency map of the cochlea from 500 Hz to 16 kHz in half octave steps was created with the software Keyence BZ-II-Analyzer by measuring the relative distances beginning from the apex of the cochlea along the spiral [20], according to the published cochlear frequency map for the Mongolian gerbil [35]. Full-focus microscopic images in the range of the synapses of the IHCs of the resulting 11 regions from the subjacent inner spiral bundle to the nerve terminal in the supra-nuclear region including all visible synaptic ribbons were obtained using a fluorescent microscope BZ9000 (Keyence, Neu-Isenburg, Germany) with a 40× objective (0.6 numerical aperture) and visualized using an ImageJ plugin. Labeled synapses of the IHC region were counted for groups of 15 IHC, according to the matching regions of the frequency map.

All statistical analyses were performed with Statistica 14 (TIBCO Software Inc., Palo Alto, CA, USA). The GPIAS effect size was assessed by nonparametric Mann–Whitney U tests, comparing animal groups independently for both treatment conditions. ABR threshold changes (control ear, trauma ear) were assessed independently for both treatment conditions by mixed design repeated-measurement ANOVAs (repetition factor *ear* and factors *stimulation frequency* and *substance application*) or two-factorial ANOVAs (factors *stimulation frequency* and *substance application*). The obtained numbers of synapses were evaluated by nonparametric Kruskal–Wallis ANOVAs separately for differently treated animal groups, as well as for trauma and control side counts.

## 3. Results

### 3.1. Hearing and Hearing Loss

**Figure 3:**
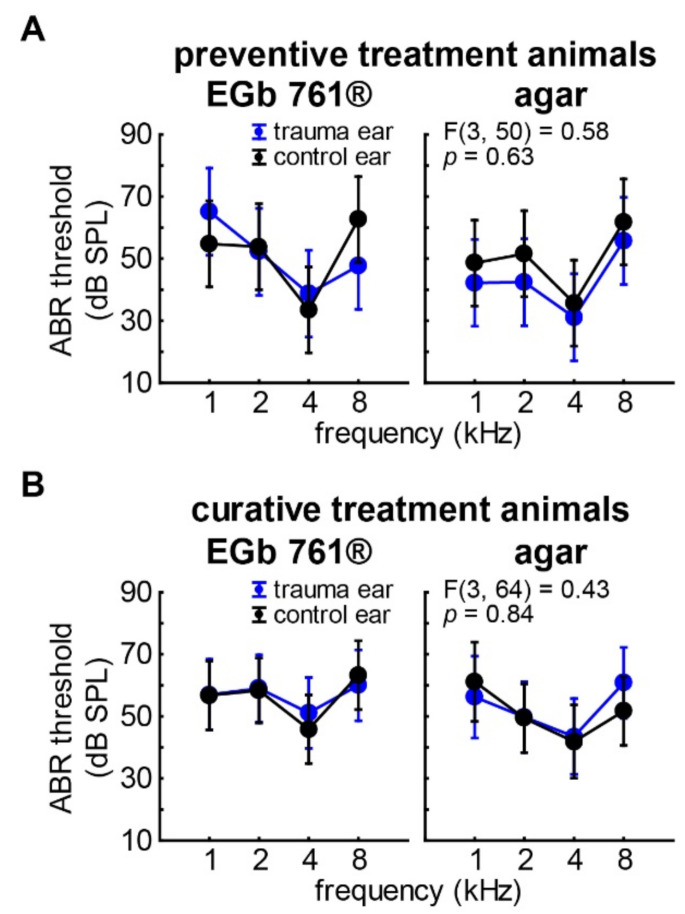
Hearing thresholds in healthy animals before acoustic trauma. (**A**) Results of the repeated-measurement ANOVA (factor *ear*: trauma ear (i.e., ear that was later traumatized) = blue, control ear = black) interaction plot (factors *frequency*: 1, 2, 4, 8 kHz; *application group*: EGb 761, agar) of hearing thresholds (dB SPL) of the 16 healthy animals in the *preventive treatment* group. Given are the mean values as filled circles and the 95% confidence intervals as whiskers, as well as the F-statistics of the interaction of all three factors on the ABR thresholds as an overview. (**B**) Results of the repeated-measurement ANOVA of hearing thresholds of the 21 healthy animals in the *curative treatment* group. For details of ANOVAs, see Table 1.

To ensure that all animal groups started from similar hearing threshold baselines, we first compared the hearing thresholds in the healthy animals, i.e., before acoustic trauma. To that end, we performed mixed design repeated-measurement ANOVAs (repetition factor *ear* and factors *stimulation frequency* and *substance application*) for both experimental approach groups (preventive and curative). An overview of the results of these analyses is given in Table 1 and depicted in the interaction plots in Figure 3. Briefly, the only significant effect found in both experimental approach groups (preventive: *p* < 0.001; curative: *p* = 0.02) was the dependency of the hearing thresholds on the stimulation frequency, i.e., the audiogram of the animals with a best hearing frequency of 4 kHz. Future control and trauma ears, as well as future application groups did not show any significant differences, even though the variance in the animals to be preventively treated (Figure 3A) was somewhat higher than in those to be curatively treated (Figure 3B), an observation which could be due to the difference in absolute animal numbers (16 vs. 21 animals).

**Figure 4:**
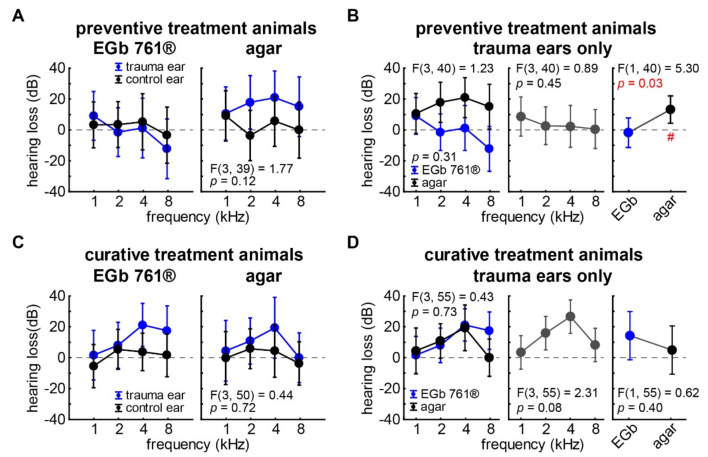
Hearing loss 3 weeks after trauma. (**A**) Results of the repeated-measurement ANOVA (factor *ear*: trauma ear = blue, control ear = black) interaction plot (factors *frequency*: 1, 2, 4, 8 kHz; *application group*: EGb 761^®^, agar) of hearing loss (dB) of the 16 animals in the *preventive treatment* group. Given are the mean values as filled circles and the 95% confidence intervals as whiskers, as well as the F-statistics of the interaction of all three factors on the ABR thresholds. For details of this ANOVA, see Table 2. (**B**) Results of the two-factorial ANOVA (*frequency* and *application group*) of the *preventive animals’* trauma ears HL only. The # symbol indicates a significant single sample *t*-test vs. zero. (**C**) Results of the repeated-measurement ANOVA of hearing loss of the 21 animals in the *curative treatment* group. For details of this ANOVA, see Table 2. (**D**) Results of the two-factorial ANOVA (*frequency* and *application group*) of the *curative animals’* trauma ears HL only.

Figure 4 and Table 2 depict the hearing threshold changes (hearing loss, HL) 3 weeks after the trauma. Again, mixed design repeated-measurement ANOVAs (repetition factor ear and factors stimulation frequency and substance application) for both experimental approach groups were calculated. In the case of the preventive treatment, we did not find any significant effects in the HL which can also be seen in the nonsignificant interaction of all factors (*p* = 0.12) in Figure 4A. Only the trauma ears of the agar-treated animals showed a significant increase in mean hearing thresholds (Figure 4B; HL averaged over all frequencies: 14.3 ± 13.7 dB; single-sample *t*-test vs. 0, *p* = 0.03), while the HL of the EGb 761^®^-treated animals did not differ from zero. In the curatively treated animals, we saw a significant overall HL in the trauma ears (9.8 ± 7.6 dB) of the curatively treated animals compared to their control ears (2.0 ± 6.3 dB; F(1,50) = 4.17, *p* = 0.03; Table 2). Nevertheless, we did not see any other significant effects or interactions on HL (Figure 4C), indicating that a curative treatment with EGb 761^®^ has no significant benefit on the hearing threshold development after trauma. This is also depicted in Figure 4D, where neither the frequency nor the substance application showed any significant effects on the HL of the trauma ears.

### 3.2. Behavioral Evidences of Tinnitus Percepts

**Figure 5:**
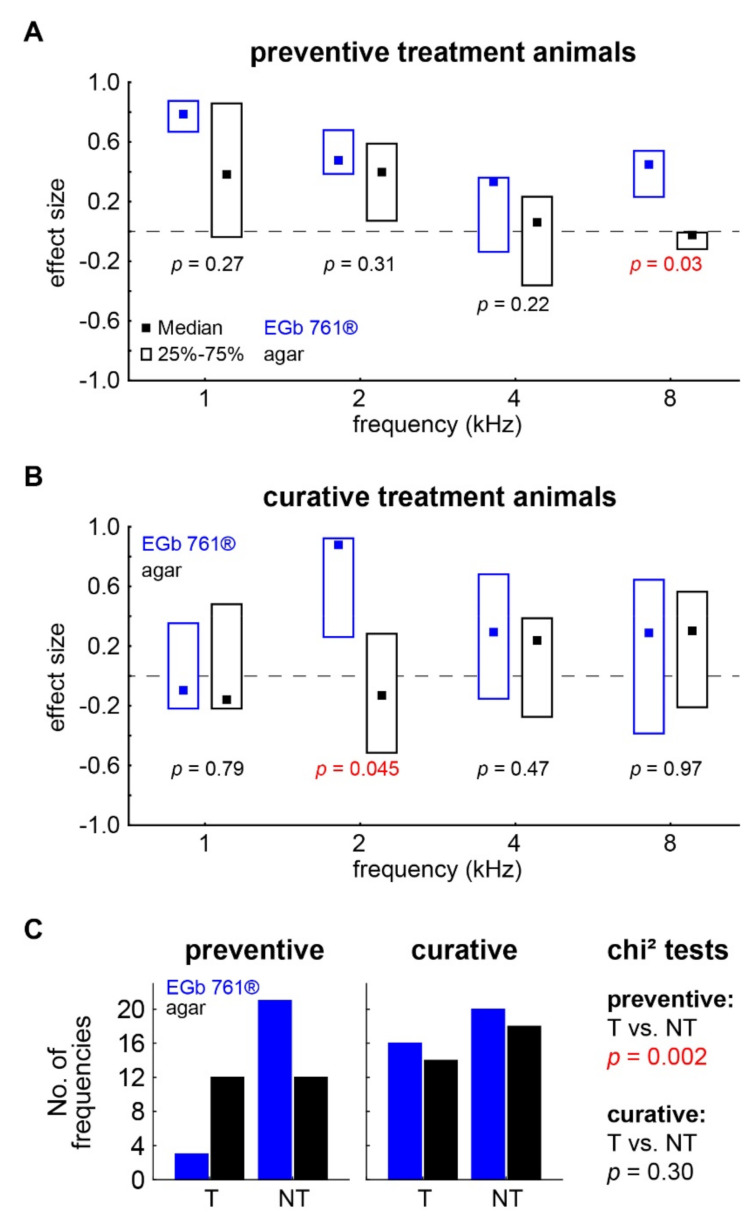
Effect size of GPIAS response change 3 weeks after trauma. (**A**) Median and interquartile ranges of effect size of behavioral response change after *preventive treatment* in EGb 761 (blue symbols) and agar control animals (black symbols) over the four tested frequencies. The *p*-values give the results of the independent Mann–Whitney U-tests. (**B**) Median and interquartile ranges of behavioral response change after *curative treatment*. (**C**) Number of frequencies indicating a tinnitus percept (T; negative effect size) or no tinnitus percept (NT) in EGb 761 and agar control animals of both treatment groups. The results of the independent chi^2^ tests T vs. NT are given on the right.

As HL is considered one of the most important causes for the development of tinnitus, we next investigated if the two different treatment approaches are able to change the likelihood of such a development. To this end, we compared baseline GPIAS behavior in the healthy animal with the behavior 3 weeks after the acoustic trauma. The difference was quantified by means of the effect size (see Section 2), with negative effect size values indicating a reduction in PPI, which in turn is believed to indicate a possible tinnitus percept. Positive effect size values, on the other hand, showed an increase in PPI not indicating tinnitus but may be a correlate of cortical learning effects (see Section 4). When comparing the effect size values of preventively EGb 761^®^-treated animals with their agar-treated control counterparts (Figure 5A), we found a general trend toward positive values especially in the lower frequency ranges of 1 and 2 kHz with no significant difference between both animal groups (Mann–Whitney U tests, *p* = 0.27 and *p* = 0.31). At 4 kHz, the effect size values were found to be more centered around zero, while also showing no significant differences between the animal groups (*p* = 0.22). By contrast, at 8 kHz, both groups showed significantly different effect size values with positive values in the EGb 761^®^ animals (i.e., no tinnitus) and more negative values (i.e., tinnitus) in the agar control animals (*p* = 0.03). With respect to the individual animals (see Figure 5C), the agar-treated animals showed behavioral evidence of tinnitus in the frequency ranges of 4 and 8 kHz, while preventively EGb 761^®^-treated animals showed tinnitus only at 4 kHz and only in isolated cases.

In the curatively treated animals, the effect size values were generally more centered around zero (Figure 5B) with most frequencies not showing differences between both groups (Mann–Whitney U tests: 1 kHz: *p* = 0.79; 4 kHz: *p* = 0.47; 8 kHz: *p* = 0.97) except for the 2 kHz frequency range, where EGb 761^®^-treated animals showed significantly stronger positive values than agar-treated control animals (*p* = 0.045). In other words, the distribution of tinnitus-related responses in agar-treated animals was broad, while curatively EGb 761^®^-treated animals showed only at 2 kHz less behavioral evidence of a tinnitus percept.

The results of the absolute effect size values were supported by the number of cases, where the individual animals showed negative (tinnitus, T) or positive (non-tinnitus, NT) effect size values (Figure 5C). We found significantly less T frequencies in preventively treated animals (chi^2^ test, *p* = 0.002) compared to their agar-treated controls but no difference in the affected number of frequencies in the curative treatment protocol. On the level of the animal, we found similar—albeit nonsignificant due to low individual numbers—results as only two preventively treated animals and five agar-treated animals showed behavioral signs of tinnitus (chi^2^ test, *p* = 0.13). Curatively treated animals with and without substance indicated behavioral signs of tinnitus in four and five cases, respectively (chi^2^ test, *p* = 0.53). In other words, on the level of single frequencies the lack of HL found in the preventively treated animals (cf. above) coincided with tinnitus development, while that in curatively treated animals HL was not prevented, and tinnitus percepts developed as frequently as in agar controls.

### 3.3. Changes in Ribbon Synapse Numbers at the IHC of the Cochlea

**Figure 6:**
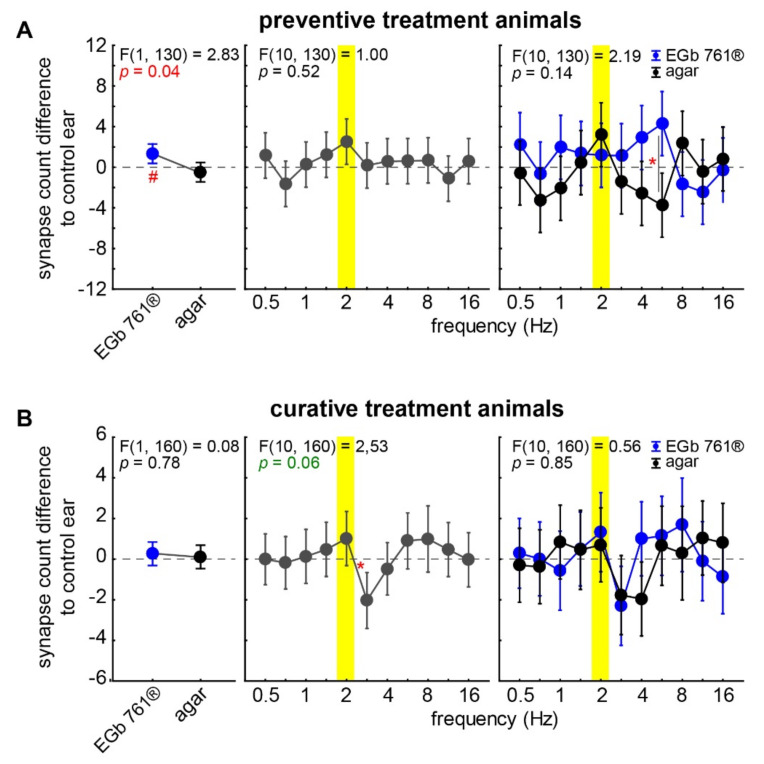
Synapse count differences of trauma and control ear (negative values show a loss of synapses) 3 weeks after trauma. (**A**) Results of the two-factorial ANOVA of the synapse difference with the factors *frequency* and *application group* in the *preventive treatment* animal group. The left and center panels give the one-factorial analyses, and the right panel depicts the interaction of both factors. The yellow bar marks the trauma frequency. Symbols depict the mean values and 95% confidence intervals of EGb 761^®^ (blue) and agar control groups (black). The F-statistic is given in the panels, where red asterisks mark significant Tukey post hoc tests (* *p* < 0.05). A red # symbol marks a significant difference from zero in the single sample *t*-test (# *p* < 0.05). (**B**) Results of the two-factorial ANOVA of synapse difference in the *curative treatment* animal group.

As we could show earlier [20], tinnitus development is strongly associated with a synaptopathy of the ribbon synapses of the IHCs and less correlated with HL itself. Therefore, we also evaluated the number of IHC synapses at 11 cochlear frequency locations of both ears in all animals and calculated the differences in synapse counts in the trauma ear relative to that of the control ear in the same individual. Negative values indicate a lower number of synapses on the trauma side (i.e., trauma led to synapse loss), whereas positive values indicate a lower number on the control side. For statistical testing, two-factorial ANOVAs (factors *stimulation frequency* and *substance application*) for both treatment approaches were calculated independently (Figure 6).

In the case of the preventive EGb 761^®^ treatment (Figure 6A), we found a significant difference in mean synapse numbers across all frequency ranges on the trauma side of substance-treated animals compared to agar-treated animals’ cochleae (F(1, 130) = 2.83, *p* = 0.04). Surprisingly, this difference was a significant increase from zero (1.7 ± 1.1 synapses/IHC, single sample *t*-test vs. 0, *p* = 0.03). While no significant frequency dependency of synapse count difference averaged across both groups was found, it became clear in the interaction plot (Figure 6A, right panel) that this was the case because the effects of EGb 761^®^ application and agar control canceled each other out. Even though the interaction did not become significant (*p* = 0.14), a Tukey post hoc test revealed a significant difference of synapse numbers at 5.6 kHz, with a significant increase in synapse numbers in the EGb 761^®^-treated animals and a decrease in synapse numbers in the agar-treated controls. In other words, the preventive treatment with EGb 761^®^ not only protected the animals from a loss of synapses in this frequency range but even enabled them to increase the synapse numbers significantly over the course of 3 weeks, specifically in the trauma-affected ear.

In the curatively treated animals’ cochleae, no overall changes in synapse numbers could be observed (Figure 6B, left panel). This seems particularly interesting, as the synapse count difference averaged over both groups did show a tendency (*p* = 0.06) for a frequency dependency. In other words, although there was no change in absolute numbers of synapses across the whole cochlea, we observed local changes in certain frequency regions, e.g., the trauma region. Consequently, synapse losses in the trauma region must have been overcompensated for by increased synapse counts in frequency regions adjacent to the trauma region (Figure 6B, middle panel). This tendency was due to the fact that, in both groups, the reduction in synapses on the trauma side was comparable (*p* = 0.85) and centered around 2.8 kHz, i.e., half an octave above the trauma frequency.

## 4. Discussion

In this study, we investigated the preventive and curative effects of the *Ginkgo biloba* extract EGb 761^®^ on hearing loss, tinnitus development, and IHC synapse numbers in Mongolian gerbils. We found that, if the extract was given orally over 3 weeks prior to an acoustic trauma, hearing loss and, consequently, the development of tinnitus were significantly reduced when investigated 3 weeks after the trauma. Even more stunning was the finding that the number of ribbon synapses in the affected ear was also significantly increased, especially in frequency ranges just above the trauma frequency. None of the described effects were significant in animals that received the extract during the 3 weeks after the trauma.

We recently published a study showing that tinnitus in Mongolian gerbils is more related to synaptopathy than to overt or hidden hearing loss [20]. Specifically, a mean loss of up to five synapses per IHC above the trauma frequency—peaking around one octave above the trauma frequency—was strongly related to the tinnitus percept but much weaker to the extent of the HL. With this background knowledge, we specifically asked whether this synaptopathy could be prevented or treated by EGb 761^®^.

Effects of *Ginkgo biloba* extracts on tinnitus were described earlier by our group [26,27] and many others (e.g., [36,37,38]) and were also summarized and discussed in a recent review [11]. Briefly, in our earlier animal study regarding the preventive effects of EGb 761^®^ [26], we found that, after 2 weeks of oral application of the extract, the noise-induced hearing loss and the development of a tinnitus percept were significantly reduced already 1 week after the binaural acoustic trauma. Additionally, we found an influence of the preventive application on the neuronal activity in the primary auditory cortex, possibly adding to the prevention of tinnitus development. This is in line with the findings in this study, even though the trauma and some measurements were slightly different. The main outcome here was that no decrease in ribbon synapse count at the IHCs could be detected in the preventively treated animals. To our surprise, even the contrary was true, and we observed a significant increase in the number of synapses in the trauma ear when compared to their intraindividual control ears. The significant increase in numbers was most prominent at the 5.6 kHz cochlear location but could also be seen in a significant general increase of 1.7 synapses per IHC, which is equivalent to a roughly 8% increase (Figure 6A). This neural growth-like effect could be possibly explained by an extract-induced decrease in apoptosis [39,40] specifically affecting the trauma ear but less so the control ear. This seems highly unlikely, as we found completely normal numbers of ribbon synapses on the control side, ranging from 20 to 24 per IHC dependent on the cochlear frequency location, and, as the extract was given orally, it should have affected both ears equally. On the other hand, it has been shown that EGb 761^®^ promotes the release of neurotransmitters such as dopamine within the central nervous system and in the cochlea [41], where dopamine release in particular might restore cochlear hair cell function [42]. This increase in neurotransmitter release might lead to the development of new synapses, especially at the affected side where neuronal activity is disturbed and neuroplasticity might be induced; a study with neuronal stem cells might support this view [43]. Whether the increase in ribbon synapse numbers at the IHCs is permanent or “normalizes” after some time has to be investigated in further studies.

In an earlier animal study regarding the therapeutic effects of EGb 761^®^ [27], we found—contrary to the findings in this report—a reduction in tinnitus-related behavioral changes over the course of the 3 weeks of application that reemerged 1 week after the end of the oral application. The hearing loss 4 weeks after the binaural acoustic trauma was significantly reduced compared to the acute hearing impairment. The results presented here are not completely in line with these findings, even though we found unimpaired behavioral responses in some frequency ranges in curatively treated animals (Figure 5B). This could have been due to the different handling of the animals, as, in the earlier study, animals were investigated regularly once a week with ABR and GPIAS experiments in addition to the daily oral application of EGb 761^®^. They also received a binaural acoustic trauma, which in turn could lead to subtle differences in neuronal plasticity-related changes leading to the development of a tinnitus percept. Lastly, the animals came from a different breeder (Charles River vs. Janvier) which may have led to a different genetic and/or physiological background of the animals. The therapeutic effects of *Ginkgo biloba* extract EGb 761^®^ on hearing loss described by other groups, e.g., in female rats [36], were comparable to our earlier study and may have been based on the oxidative stress-reducing and/or anti-inflammatory qualities of the extract [37,44], or due to the increase in cochlear blood flow optimizing oxygen perfusion of the tissues (e.g., [45]). Nevertheless, we did not see an overall effect of the extract on the number of surviving ribbon-synapses at the IHCs of therapeutically treated animals when compared to control animals (Figure 6B). Interestingly, the finding that, despite the lack of overall changes in synapse counts in the curative regime, we saw a tendency to local differences in synapse counts points to an overcompensation for synapse loss in the trauma region by adjacent regions. This observation will have to be further evaluated in future studies.

The behavioral method used for the identification of a possible tinnitus percept was the reflex-based GPIAS measurement. The validity and strength of the method is still under debate [46,47,48] but has been improved from its beginnings [33] over time (e.g., [30]). We always combine the results of the GPIAS paradigm with physiological results such as hearing loss, which should be—according to our hypothesis—correlated with the strength of the tinnitus, i.e., the effect size. In a recent publication, we were able to show exactly this correlation [31], supporting the validity of the method. In line with our animal-experiment tested hypothesis [8,9,20,22,49,50], as well as with human patient studies and treatment approaches [18,19,51], these animals, therefore, do show behavioral signs of tinnitus. Independent of the details, in this study, a clear difference between preventively and curatively treated animals and their respective controls emerged. While preventively treated animals hardly showed any signs of tinnitus, the curatively treated animals were not different from controls, showing roughly 50% of the frequencies being affected, at least to a certain degree. As the preventively treated animals did not receive the extract after the acoustic trauma, and as the time of the GPIAS tinnitus test relative to the last application of the extract was 3 weeks, a possible acute anxiety-releasing or mood-enhancing effect of EGb 761^®^ [52] on the animals can be ruled out.

Taken together, the preventive effect of EGb 761^®^ against hearing loss and tinnitus in our animal model was seemingly based on increased numbers of ribbon synapses at the IHCs of the cochlea. Hardly any curative effect of the extract could be found in this animal model, but this may be a subject for further investigation, as the literature suggests that hearing loss and tinnitus might be reduced under the correct conditions.

## Figures and Tables

**Table 1:** Results of repeated measurement ANOVAs of healthy animals’ hearing thresholds in *preventive* and *curative effect* groups.

Treatment	Factor	F-Statistics	*p*-Value
**Preventive**	Ear	F(1, 50) = 0.91	0.34
	Frequency	F(3, 50) = 8.45	<0.001
	Substance	F(1, 50) = 2.19	0.15
	Ear × frequency	F(3, 50) = 0.64	0.59
	Ear × substance	F(1, 50) = 0.79	0.38
	Frequency × substance	F(3, 50) = 1.30	0.29
	Ear × frequency × substance	F(3, 50) = 0.58	0.63
**Curative**	Ear	F(1, 64) = 1.41	0.24
	Frequency	F(3, 64) = 3.85	0.02
	Substance	F(1, 64) = 1.11	0.30
	Ear × frequency	F(3, 64) = 1.30	0.28
	Ear × substance	F(1, 64) = 0.72	0.40
	Frequency × substance	F(3, 64) = 0.49	0.69
	Ear × frequency × substance	F(3, 64) = 0.43	0.84

**Table 2:** Results of repeated-measurement ANOVAs of animals’ hearing loss in *preventive* and *curative effect* groups.

Treatment	Factor	F-Statistics	*p*-Value
**Preventive**	Ear	F(1, 39) = 0.46	0.50
	Frequency	F(3, 39) = 1.39	0.21
	Substance	F(1, 39) = 0.03	0.87
	Ear × frequency	F(3, 39) = 0.16	0.92
	Ear × substance	F(1, 39) = 0.48	0.48
	Frequency × substance	F(3, 39) = 0.92	0.44
	Ear × frequency × substance	F(3, 39) = 1.77	0.12
**Curative**	Ear	F(1, 50) = 4.17	0.03
	Frequency	F(3, 50) = 0.67	0.57
	Substance	F(1, 50) = 0.01	0.93
	Ear × frequency	F(3, 50) = 0.31	0.82
	Ear × substance	F(1, 50) = 0.82	0.37
	Frequency × substance	F(3, 50) = 0.65	0.59
	Ear × frequency × substance	F(3, 50) = 0.44	0.72

## Data Availability

The data supporting the findings of this study are available upon request to the corresponding author.
